# Bicycling for Women

**Published:** 1895-09

**Authors:** 


### Bicycling for Women.

The much discussed question as to whether bicycling is per se an exercise suited to women is, perhaps, now of minor importance, since it has become fashionable, and will, consequently, be adopted by the gentler sex, without much regard as to its immediate or remote effects. Its popularity is, however, a very satisfactory proof as to its capacity as a giver of health and pleasure, since it is not conceivable that the bicycle could ever have won its way, as did the corset, for instance, as a fancied adjuvant to physical charms. The late Dr. William Goodell, wise in an unusually wide experience, gave the exercise his unqualified indorsement, and from the almost entire absence of adverse criticism, it is apparent that the majority of gynecologists are of the same mind. The whole question is an exceedingly simple one, and has been summed up by Dickenson (*Amer. Jour. of Obstet.*), who concludes a very clear and complete paper upon this subject as follows:

Under proper conditions of costume and posture, with care that the exercise be gradually increased and properly graded for the individual case, and where there is no acute inflammation to contra-indicate it, bicycling will probably show itself capable of large results as an agent in curing pelvic disorders, since it is one of the few exercises which attract women.

In view of woman's disabilities and the disadvantages under which she has suffered in attempts to obtain interesting and beneficial muscular exercise, it seems hardly too much to say that the promise from the bicycle is far-reaching. Through it and the habits it will engender we look for better dress, freer dress, shorter dress in bad weather; for better exercise, for out-door activity, for steadier nerves, stronger muscles, painless periods, easy labors.—*Medical Reporter.*

**Death-bed Repentance.**—In our August issue the title of Dr. Bennett's paper was spelled "hemerrhoids" instead of "hemorrhoids;" error not discovered until too late to correct it. They say, it is never too late to mend; we hope it is not too late to make amends.

